# Repurposing Antidiabetic Medications for Parkinson’s Disease: Focus on Biomarker Strategies for Disease Modification

**DOI:** 10.3390/ijms27062560

**Published:** 2026-03-11

**Authors:** Narayana K. Yelleswarapu, Christos Sidiropoulos

**Affiliations:** Department of Neurology and Ophthalmology, Michigan State University, East Lansing, MI 48824, USA; sidirop3@msu.edu

**Keywords:** Parkinson’s disease, antidiabetic medications, biochemical biomarkers, disease-modifying therapies

## Abstract

Parkinson’s disease (PD) is a progressive neurodegenerative disorder. It shares many pathophysiologic similarities with type 2 diabetes mellitus (T2DM). Numerous studies have explored the repurposing of antidiabetic medications for their potential neuroprotective effects in PD. There has not been a consolidated review of the biochemical biomarkers that have been evaluated across antidiabetic medications. This review aims to assess the current landscape of biomarker research in evaluating the efficacy of these antidiabetic agents as disease-modifying therapies in PD. We examine the molecular mechanisms targeted by these drugs, the biomarkers used to assess their effects, and the outcomes of clinical trials. This review hopes to identify gaps in current research and enhance the evaluation of antidiabetic medications in PD.

## 1. Introduction

Parkinson’s disease (PD) is a progressive neurodegenerative disorder characterized by loss of dopaminergic neurons within the substantia nigra thought to be predominantly caused by accumulation of α-synuclein (αSyn) pathology. Gradual depletion of dopaminergic neurons results in impaired motor activity, resulting in resting tremor, bradykinesia, rigidity, and postural instability [1]. In addition, non-motor symptoms, i.e., mood disorders, dysautonomia, sleep disturbances, cognitive impairment, and gastrointestinal complications, can occur up to 20 years preceding motor manifestation of the disease [2]. Despite tremendous advances in symptomatic treatment over the last 50 years, there are currently no FDA-approved disease-modifying therapies capable of slowing or halting neurodegeneration. PD continues to remain incurable despite significant progress made in its management.

Over the years, there has been a focus on advancing diagnostic testing to better diagnose PD early, accurately, and to initiate appropriate treatment. The landscape has included tests such as dopamine transporter imaging (DaTscan), olfactory tests, polysomnography, gastric emptying assay, αSyn skin biopsy, cerebrospinal fluid (CSF) αSyn seed amplification assay, and genetic testing [3,4]. In conjunction, there has been development in biochemical biomarkers that facilitate the early diagnosis of PD. Repurposing therapies that target these biochemical biomarkers may help not only with earlier diagnosis but also with better outcomes for PD patients.

There has been an emerging link between type 2 diabetes mellitus (T2DM) and PD. T2DM is a metabolic disease defined by insulin resistance and hyperglycemia. While distinct in their primary clinical manifestations, increasing evidence suggests that metabolic dysfunction, impaired insulin signaling, oxidative stress, blood–brain barrier dysfunction, and mitochondrial dysregulation contribute to PD pathophysiology, linking central nervous system degeneration to systemic metabolic disturbances [4,5]. Accordingly, antidiabetic medications have shown neuroprotective effects in preclinical and early human studies. Therefore, there has been an increasing interest in translational research looking at how antidiabetic medication alters biomarkers of neurodegeneration in PD.

In this review, we will explore the recent advances in antidiabetic medications that have been linked with disease modification on key PD biomarkers and their proposed outcomes in preclinical and clinical models of PD.

## 2. Relationship Between T2DM and PD

T2DM has been shown to increase the risk of developing PD and accelerate its progression. In multiple meta-analyses, individuals with T2DM were associated with increased risk for PD, with relative risk ranging from 1.19 to 1.27 [6,7,8]. Cohort studies from Korea have shown that T2DM duration was associated with higher PD risk [9]. Another large cohort study from the UK has shown that there were elevated rates of PD following T2DM (hazard ratio 1.32, 95% confidence interval 1.29–1.35; *p* < 0.001) and that the increase was greater in individuals with complicated T2DM [10]. Table 1 summarizes the relationship and interplay between these two diseases.

Mechanistically, there is extensive literature that shows pathophysiological similarities between T2DM and PD. First, insulin resistance and dysregulated insulin signaling are central to both diseases. In T2DM, misfolding of amylin may contribute to pathology due to loss of pancreatic beta-cells, which in turn, leads to peripheral insulin resistance and then leads to hyperglycemia and β-cell dysfunction [11]. Amylin has been suggested to be involved in PD by interacting with αSyn, with evidence of coexistence of these aggregates in both serum and brain-derived extracellular vesicles in PD patients [12]. In PD, insulin resistance occurs in the central nervous system and contributes to dopaminergic neuronal degeneration [11,13,14]. Insulin is thought to influence nervous system development, acts as a neuroprotector by controlling apoptosis and ischemia [15], and insulin metabolism impairment is thought to lead to cognitive impairment and increase the risk of neurodegenerative disorders. Vice versa, dopaminergic activity in the CNS appears to modulate peripheral insulin-mediated glucose metabolism, as in vivo studies have shown dopaminergic drugs influence insulin production, insulin resistance, and glycemic control [16]. Moreover, insulin-like growth factor-1 (IGF-1), a hormone with structural similarity to insulin and involved with cell growth, has been shown to have an inverse association with T2DM and PD. Low IGF-1 levels have been associated with a higher risk of diabetes through increased insulin resistance and β-cell dysfunction [17,18], whereas higher levels of IGF-1 have been found in PD patients [19,20,21]. This suggests that IGF-1 has different roles, where peripherally it is involved in maintaining glucose homeostasis and centrally it is involved in cell survival and may have neuroprotective effects.

Secondly, chronic inflammation and activation of the nuclear factor kappa B (NF-κB)/nod-like receptor pyrin 3 (NLRP3) inflammasome axis are implicated in both T2DM, where hyperglycemia-induced oxidative stress triggers systemic inflammation, and in PD, where neuroinflammation is enhanced, leading to increased αSyn aggregation and further driving dopamine neuron loss [22,23,24]. NF-κB is a DNA-binding protein required for transcription of many proinflammatory cytokines and chemokines. NF-κB is activated by reactive oxygen species (ROS) and proinflammatory cytokines, thus chronic low-grade inflammatory disorders such as T2DM result in higher levels of activated NF-κB [25]. In PD, NF-κB is thought to cause pathogenesis through the induction of inflammation-mediated degeneration of dopaminergic neurons, and this has been demonstrated in a mouse model, where inhibiting NF-κB prevented degeneration of dopaminergic neurons [26]. αSyn can also trigger activation of NF-κB and further release proinflammatory cytokines, which further damage dopaminergic neurons [27]. NLRP3 inflammasome is involved with the activation of caspase-1 and maturation of IL-1β and IL-18, which trigger and coordinate the release of cytokines. In T2DM, NLRP3 inflammasome functions as a sensor for metabolic stress [28], can reduce glucose tolerance through modulation of gut microbiota [29], and may induce pancreatic β-cell pyroptosis [30]. In PD, the NLRP3 inflammasome can also cause degeneration of dopaminergic neurons by induction of pyroptosis, which can cause αSyn aggregation [31] but also further inflammatory responses by being triggered by αSyn aggregation [32,33]. Connecting T2DM to PD, chronic hyperglycemia is shown to increase αSyn deposition in pancreatic β-cells and trigger degeneration of dopaminergic neurons in PD [34].

Thirdly, both T2DM and PD have shown mitochondrial dysfunction, ferroptosis, and oxidative stress, especially in β-cells and dopaminergic neurons, which lead to cell death and disease progression [23,24,35]. Mitochondrial dysfunction in T2DM is thought to be a result of decreased ATP levels, likely secondary to insulin dysfunction and increased vulnerability to oxidative stress [36]. PD is a disease that affects the aging brain, and aging results in mitochondrial dysfunction [37]. Specifically, loss of the mitochondrial complex I causes cell death in dopaminergic neurons, and this has been observed in mouse models in T2DM [38,39].

Fourthly, both T2DM and PD show similar patterns of impaired protein handling and aggregation. T2DM is characterized by islet amyloid polypeptide (IAPP) aggregation, while PD features αSyn aggregation. IAPP is a 37-amino acid peptide produced by pancreatic β-cells in response to hyperglycemia. When IAPP aggregates and forms fibrils, it disrupts β-cell function. Studies have shown that αSyn promotes IAPP aggregation [40]. On the contrary, evidence in animal models, including mice and cynomolgus monkeys, shows that IAPP can act as a trigger for αSyn pathology and accelerates its accumulation and neurotoxicity in dopaminergic neurons [41,42,43]. Speculation is that both proteins interact with one another in the pancreas as well as within the CNS after blood–brain barrier breakdown in elderly populations.

## 3. Biochemical Biomarkers in PD

Vijiaratnam et al. define a biomarker as “an objectively measured and evaluated characteristic from any substance, structure or process that can be measured in the body or its products as an indicator of normal biological or pathogenic processes, or pharmacologic responses to a therapeutic intervention” [44]. Therefore, an ideal biomarker should be reliable across the population and accessible through clinical samples. Reliable biomarkers become useful when assessing disease progression and response to interventions.

In PD, the landscape of biomarker analysis is vast and includes clinical, imaging, genetic, and biochemical markers. For the purpose of this review, we will focus on the biochemical biomarkers that have been involved in the pathogenesis of PD. Biochemical biomarkers are collected from body fluids or tissue biopsies. Four classes of potential biochemical biomarkers that have been studied in PD include αSyn, markers of neuroinflammation, markers of insulin resistance, and markers of synaptic degeneration.

In PD, one of the earliest biomarkers that was evaluated was αSyn, the pathological protein that has been implicated in the substantia nigra, resulting in dopaminergic neurodegeneration. Tests, evaluating total, phosphorylated, and oligomeric αSyn in CSF, plasma, saliva, and skin samples have been developed [45]. CSF αSyn seeding amplification assay (SAA) is highly accurate in differentiating PD from healthy controls when studied in a large and well-characterized cohort sample, but showed variability in the LRRK2 variant population [46]. Commonly used in neurology clinics, the skin biopsy detection assay detected cutaneous phosphorylated αSyn with high accuracy in patients with PD and PD plus syndromes, making this a minimally invasive technique [47]. Moreover, phosphorylated αSyn, specifically Ser-129p-αSyn, appears to be most specific for disease presence and severity. Unfortunately, none of the αSyn samples have been able to predict disease progression [44,48,49].

Markers of neuroinflammation (GFAP, complement levels, TNF, CRP, and interleukin levels), including mitochondrial markers (DJ-1 and PPAR-γ), and lysosomal dysfunction (glucocerebrosidase activity, β-hexosaminidase, and cathepsin D) have shown mixed results and have not been consistent in differentiating from atypical PD syndromes and measuring progression of PD [44]. In addition, inflammatory cytokine levels are elevated in a wide range of conditions.

Due to the increased risk of PD in T2DM patients, markers of insulin resistance have been considered [50]. Specifically, elevated insulin-receptor substrate-1 (IRS-1) phosphorylation, representing attenuated insulin signaling, has been found in PD patients with worsening tremor [51]. Another marker of insulin resistance is increasing hemoglobin A1c levels. Elevated hemoglobin A1c levels were shown to be associated with motor and cognitive severity and axonal damage in PD [52,53]. As discussed earlier, plasma IAPP levels can be an indirect measure of IAPP deposition within the CNS, which can implicate the burden of αSyn aggregation and disease burden in PD [54].

Markers of synaptic degeneration (SNAP25, β-synuclein, GAP43, and Contactin-1) and axonal damage (NfL) have shown moderate predictability of severity of motor and cognitive progression when combined with clinical and disease-specific fluid biomarkers [44,55]. Specifically, NfL shows promise for discriminating between PD and PD plus syndromes [55,56].

Several of these biochemical biomarkers associated with PD are affected by antidiabetic medications [45].

## 4. Antidiabetic Medications and Their Interactions on PD Biomarkers

### 4.1. Biguanides

Metformin, a drug from the biguanide class, is a relatively low-cost medication with minimal side effects, which makes it the preferred first-line diabetic treatment worldwide. Metformin stabilizes an individual’s glycemia by inhibiting hepatic gluconeogenesis, promoting peripheral glucose uptake, and increasing insulin sensitivity. Multiple studies from cellular and animal PD models have shown that metformin improves lifespan, reduces dopaminergic neuron loss, and improves motor deficit recovery [57].

Preclinically, these neuroprotective effects are thought to be predominantly produced by activation of autophagy via AMPK signaling, which promotes degradation of misfolded proteins and damaged organelles [57,58,59]. This, in turn, can inhibit αSyn phosphorylation at Ser129 and reduce accumulation by directly interacting with αSyn monomers [57,60,61]. In MPTP mouse models, metformin use has shown reductions in oxidative stress and mitochondrial dysfunction, thus improving neuronal survival and function [57,62,63]. Metformin use has also been shown to upregulate neurotrophic factors such as brain-derived neurotrophic factor (BDNF) and glial cell-line derived neurotrophic factor (GDNF), which support dopaminergic neuron health [60,64,65], while suppressing elevated levels of neuroinflammatory markers and glial activation in PD [63,66]. However, metformin appears to also have neuroprotective effects independent of AMPK activation, as shown in a study with MPTP-induced AMPK knock-out mice treated with metformin [66].

Clinical outcomes focusing on improvement in motor and cognitive impairment showed mixed benefits [67,68,69]. However, a recent randomized pilot study found that there was no significant difference in motor outcomes between the metformin and control groups after treatment, though metformin significantly reduced neuroinflammatory and pathological biomarkers [64]. Future clinical trials will need to address different doses of metformin and its effect on αSyn detected through CSF or through skin biopsy assays in the PD population, as well as motor or non-motor outcomes, to draw direct correlations between disease pathology, clinical status, and medication use.

However, metformin may also negatively affect PD neuropathology. In vivo and in vitro studies have found that chronic metformin use can reduce microglial activation and increase dopaminergic damage in response to MPTP [70] but also worsen cognition due to a decrease in neurotrophic factors [71]. Clinically, chronic metformin use has shown no neuroprotective effects [68].

### 4.2. Sulfonylureas

Sulfonylureas (glipizide, glimepiride, and glyburide) are another widely prescribed antidiabetic medication type typically prescribed after metformin. They act primarily by blocking ATP-sensitive potassium channels in the pancreatic beta-cells, which stimulate insulin secretion, thus lowering blood glucose.

There has not been promising data to suggest that sulfonylureas are effective at disease modification in PD [72]. One study has shown that it may even risk of PD [73]. No clinical trials of sulfonylurea use in PD were found.

### 4.3. Thiazolidinediones (TZDs)

Thiazolidinediones (pioglitazone and rosiglitazone) are a class of peroxisome proliferator-activated receptor gamma (PPAR-γ) agonists that improve insulin sensitivity and decrease blood glucose levels and are thus used for treatment in diabetes.

Preclinical data have shown potential modification of inflammatory effects through inhibition of microglia and astrocytes, as well as production of proinflammatory cytokines and nitric oxide [74]. Several in vivo PD models showed neuroprotective benefits of TZDs, specifically their role in facilitating hippocampal neurogenesis, improving dopamine survivability, and improving motor performance [75,76,77].

Clinically, retrospective cohort and meta-analysis studies have shown a potential neuroprotective effect of TZD in PD and suggest that it may even reduce the incidence and risk of PD progression [78,79,80]. A Taiwan-based large population database analysis of diabetic patients revealed that those taking TZDs had a significantly lower risk of developing PD compared to the non-TZD group in a dose-dependent manner [81]. However, in a phase 2 double blind, randomized trial, pioglitazone at 15 mg/day and 45 mg/day could not modify the progression of disease in early PD [82]. Disappointingly, there are limited studies that address the mechanism behind the benefits of TZDs in PD risk. The discrepancy between the preclinical data, meta-analyses, and clinical data has not yet indicated whether this class of diabetes medication truly holds promise in the landscape of more novel diabetic therapies.

### 4.4. Dipeptidyl Peptidase-4 Inhibitors (DPP4i)

DPP4i agents (sitagliptin, linagliptin, saxagliptin, and alogliptin) are hypoglycemic agents associated with a low risk of hypoglycemia and weight gain in patients with diabetes. They improve glucose metabolism by enhancing the bioavailability of active glucagon-like peptide-1 (GLP-1) and by inhibiting its degradation.

In vivo model studies show that DPP4i (1) upregulate neuroprotective pathways such as PI3K/AKT and Nrf2 [83], (2) increase levels of neurotropic factors such as BDNF and C-reactive element binding protein (CREB) [84,85], (3) reduce oxidative stress through indirect measurements of glutathione and malondialdehyde [85], (4) downregulate proinflammatory markers (TNF-α, IL6, Iba-1, and GFAP) and NF-κB, and reduce dopaminergic degeneration [84,86], and (5) decrease αSyn aggregation [87].

In the clinical setting, drug naïve, de novo PD patients with diabetes that were treated with DPP4i had significantly higher baseline DAT availability in the anterior, posterior, and ventral putamen compared to diabetic PD patients not on DPP4i [88]. Furthermore, this study showed that the DDP4i group had a slower increase in levodopa-equivalent dose over time and a lower rate of levodopa-induced dyskinesia.

While in vivo studies draw strong links between DPP4i use and αSyn aggregation, clinical studies have yet to explore whether these antidiabetic agents affect αSyn physiology.

### 4.5. Sodium-Glucose Cotransporter-2 Inhibitors (SGLT2i)

SGLT2i (dapagliflozin, empagliflozin, canagliflozin, ertugliflozin, ipragliflozin, tofogliflozin, and remogliflozin) are a class of antidiabetic medications that inhibit the reabsorption of glucose via the SGLT2 in the proximal tubules of the nephron and, therefore, lower blood sugar by enhancing glycosuria. Owing to their pleiotropic effects, they have also shown great efficacy for cardiovascular health, renal diseases, weight loss, and stabilizing blood pressure [89]. One of the predominant mechanisms by which SGLT2i produces these additional benefits is through the reduction in reactive oxidative species and protecting the integrity of the mitochondria [90]. Therefore, studies indicate that SGLT2i improves mitochondrial function by reducing oxidative stress, enhancing mitochondrial biogenesis, and restoring autophagic-lysosomal balance [91,92,93].

Interestingly, SGLT2 is also found in the hippocampus, cerebellum, and at the blood–brain barrier endothelial cells [94]. This makes the SGLT2i particularly intriguing CNS penetrating drugs. Preclinical studies have provided promising solutions for PD. In a rat model, rotenone was used to mimic PD-like motor deficits, and empagliflozin (SGLT2i)-treated groups showed improvements in motor function, decreases in αSyn accumulation, and decreased inflammation, which was suggestive of its neuroprotective effect [95]. Similarly, in a rotenone-induced PD zebrafish model, empagliflozin-treated groups showed improved DOPA/DA ratio, improved performance on the Y-maze task, and increased markers related to inflammation and autophagy [96]. Another rotenone-induced PD rat model study has shown attenuation of cognitive dysfunction, improvement of dopamine secretion, and decreased dopaminergic neuronal loss [97]. Multiple similar studies in toxin-induced PD models have shown improvements after SGLT2i therapy through improvements in autophagy, mitochondrial function, and inflammation [97,98,99,100]. In addition, SGLT2i agents have been shown to downregulate *Snca* gene expression in a diabetic mouse model [101].

Clinically, SGLT2i use has also shown reductions in the risk of PD dementia [102] and a lower risk of incidence of PD in T2DM populations [103,104]. In a large head-to-head comparison study between SGLT2i and metformin using 20 year dataset of almost 900,000 patients, SGLT2i was associated with a 28% lower PD risk than metformin, suggestive of its superiority in neuroprotection [105]. Another study has shown that SGLT2i were superior to DPP4i in significantly lowering risk of PD after using a retrospective cohort analysis of 89,000 Medicare beneficiaries [103]. However, this meta-analysis looking at dapagliflozin found no significant association between the medication use and risk of dementia in PD [106]. Taken together, there is promising in vivo data regarding the use of SGLT2i therapy in PD models; however, there have not been any randomized controlled trials studying this medication in PD populations.

### 4.6. Glucagon-like Peptide-1 Receptor Agonists (GLP-1 RA)

GLP-1 RA (exenatide, liraglutide, semaglutide, dulaglutide, tirzepatide, and lixisenatide) are incretin hormones that enhance glucose-induced insulin secretion and inhibit both gastric emptying and glucagon secretion [107]. Like the SGLT2i class, they have multiple pleiotropic effects, including reducing obesity, improving cardiovascular health, and improving bone health. They act via both peripheral and central mechanisms; thus, there has been a lot of interest in the GLP-1 RA and their role in PD.

In preclinical models, GLP-1/GIP dual agonist DH3-CH alone and liraglutide alone (GLP-1 RA) were both effective at reducing motor decline in the MPTP mouse model of PD. This study also found decreased expression of αSyn and activation of the Wnt/β-catenin signaling pathway, a regulatory molecule in cell proliferation, differentiation, apoptosis, neurogenesis, and stem cell maintenance [108]. Similarly, another study looking at another GLP-1/GIP dual receptor agonist, DA5-CH, in a MPTP mouse model showed reversal of MPTP-induced decrease in BDNF and GDNF, whereas it reduced αSyn and levels of inflammation and proinflammatory markers (IL6, Il-Iβ) [109]. Another study in the MPTP PD mouse model found that GLP-1 RA activated PGC-1α, which regulates autophagy and cell apoptosis, and induced a neuroprotective effect [110]. In MitoPark mice, GLP-1 RA was found to reduce tyrosine hydroxylase expression by lowering reactive oxidative species and thus decreasing dopaminergic denervation [111], and another GLP-1 RA, PT320, was shown to improve L-DOPA-induced dyskinesias [112].

Clinical trials with exenatide have shown modest improvements in motor and cognitive symptoms [113,114,115]. Lixisenatide therapy resulted in modest effects in terms of progression of motor disability in early PD [116]. Liraglutide treatment was effective at improving both motor and nonmotor symptoms in PD, with effects sustained while off medication [117]. In a population-based study, new users of GLP-1 RA have been shown to have a 23% lower risk of PD than new users of DPP4i [118]. However, a double blind randomized, placebo-controlled trial looking at exenatide over 96 weeks did not find any evidence of it acting as a disease-modifying treatment in PD patients when using MDS-UPDRS part III scores [119]. Interestingly, a recent meta-analysis of studies involving GLP-1 RA demonstrated a detrimental effect on motor outcomes compared to placebo [120]. Overall, this class of antidiabetic medication shows the most promise in PD disease modification, and multiple clinical trials are underway to draw clearer conclusions on their effects in PD.

## 5. Conclusions

**Figure 1:**
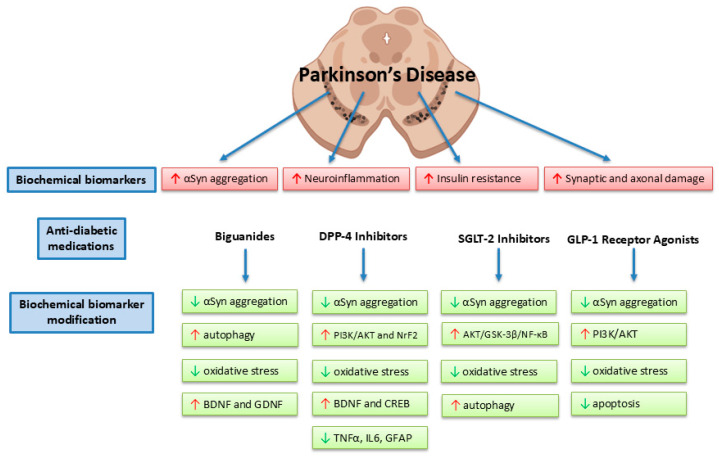
Antidiabetic medications modify metabolic, inflammatory, and neuroprotective biochemical biomarkers in Parkinson’s disease. The four classes of antidiabetic medications (biguanides, DPP4i, SGLT2i, and GLP-1 RA) have shown robust preclinical data in modifying biochemical biomarkers in PD.

There has been extensive evidence linking T2DM and PD. Specifically, molecular mechanisms responsible for disease pathology and progression in PD are thought to further their effects through insulin resistance, chronic inflammation, mitochondrial dysfunction, and impaired protein aggregation. Insulin can have a significant impact on brain function. Increasing blood glucose levels can worsen motor and cognitive performance in PD, and therefore, strategies for overall control of diabetes in the PD population can be neuroprotective. Antidiabetic medications hold promise as disease-modifying therapies in Parkinson’s disease, though much of this is isolated to preclinical model data. Antidiabetic medications modulate several biochemical biomarkers relevant to PD, summarized in Figure 1. The GLP-1 RAs show the most consistent neuroprotective properties in experimental settings. Sulfonylureas and TZDs do not show tremendous disease-modifying properties in PD.

However, there are inconsistencies in how these medications may, if at all, modify disease progression. Several clinical trials have not been able to translate the same robust findings from preclinical models. It is possible that the effect of these medications in animal models occurs as a result of the poor choice of PD models. In addition, there are far too many biomarkers being assessed as a measure of neuroprotective function, but very few have shown phenotypic improvement in human clinical trials. Overall, results from clinical outcomes in antidiabetic medications remain inconclusive.

Plausible explanations may be that the motor scales used are not sensitive enough, or non-motor outcomes should also be considered, or that the effects of those medications vary across disease stages. Another strategy might be to implement a “fit-for-purpose” biomarker validation process where the chosen biomarker is validated for its intended purpose through the generation of data and assay refinement in a continuous process [121]. For example, αSyn seed amplification assays and phosphorylated αSyn biopsies are, at this moment at least, binary, i.e., qualitative or not quantitative outcomes, which do not, yet, allow for disease progression staging, rather than perhaps detecting patients who harbor pathology and are suitable for clinical trial enrollment. Some of the patients with positive αSyn test never progress to actual disease, and, on the other hand, there are αSyn-negative patients with PD. These are the limitations for most preclinical biomarkers.

To demonstrate the neuroprotective effects of antidiabetic medications in patients with PD, we believe that clinical trials may benefit from focusing on (1) biomarker endpoints such as serum markers of oxidative stress (malondialdehyde or glutathione), neuroinflammatory markers (NF-κB), or phosphorylated αSyn in CSF, (2) specific outcome measures in addition to motor endpoints such as non-motor and digital outcome measures, (3) patient follow up over a span of 5–7 years to detect clinically meaningful differences and at appropriate phases of PD disease progression, and (4) possible consideration for stratifying populations based on genetic mutation carriers (LRRK2, SNCA, GBA1, PRKN, etc.). We have proposed a biomarker-driven clinical trial workflow for GLP-1 RA as an example in Table 2.

There is tremendous hope looking ahead, though, as more ongoing clinical trials are addressing some of these setbacks. Given the increase in focus on T2DM and weight management, the future of development and validation of biomarkers in PD is crucial for assessing the efficacy of these antidiabetic treatments, with the hope of repurposing them into effective PD neuroprotective agents.

## Figures and Tables

**Table 1:** Relationship between Parkinson’s disease and T2DM. This table focuses on the mechanistic interplay between PD and T2DM in insulin resistance, neuroinflammation, mitochondrial dysfunction, and impaired protein handling.

Mechanism	Parkinson’s Disease	Type 2 Diabetes Mellitus
Insulin resistance	Amylin may interact with αSyn and result in dopamine cell death Impaired insulin metabolism can lead to decreased neuroprotective effects	Misfolding of amylin causes pancreatic β-cell death and peripheral insulin resistance leading to hyperglycemia
Neuroinflammation	NF-κB and NLRP3 inflammasome axis lead to αSyn pathology	Metabolic stress from diabetes increases activation of NF-κB and NLRP3 inflammasome
Mitochondrial dysfunction	Loss of mitochondrial complex I results in dopaminergic cell death	Insulin dysfunction results in increased vulnerability to oxidative stress
Impaired protein handling	IAPP can trigger αSyn pathology	IAPP aggregation disrupts β-cell function αSyn can promote IAPP aggregation

**Table 2:** Proposed biomarker-driven clinical trial workflow. We have used GLP-1 RA as an example to create a workflow from patient screening to clinical outcomes with the goal of assessing for neuroprotective benefit with this medication in the PD population.

Phase	Objective	Key Biomarkers
Screening	Select homogeneous population most likely to respond to GLP-1 RA	αSyn SAA (diagnostic confirmation of disease)
Stratification	Group patient population by genotype (GBA1, LRRK2) or metabolic factors i.e., HgA1c > 6.5%, BMI > 30	
Target Engagement	Confirm drug reaches CNS and engages target	Neuronal-derived extracellular vesicles to measure IRS-1
Proof of Concept	Demonstrate effect on disease progression biomarkers	Serum reduction in NF-κB levels
Clinical Outcomes	Confirm disease modification with clinical benefit	Primary: MDS-UPDRS, cognitive scales

## Data Availability

No new data were created or analyzed in this study. Data sharing is not applicable to this article.

## Abbreviations

The following abbreviations are used in the order of appearance in the manuscript:

PD	Parkinson’s disease
T2DM	Type II Diabetes Mellitus
αSyn	α-synuclein
DaTscan	dopamine transporter imaging
CSF	Cerebrospinal fluid
IGF-1	Insulin-like growth factor-1
SAA	Seeding amplification assay
NF-κB	Nuclear factor kappa B
NLRP3	Nod-like receptor pyrin 3
IRS-1	Insulin-receptor substrate-1
AMPK	AMP-activated protein kinase
HbA1c	Hemoglobin A1c
GDNF	Glial cell-line derived neurotrophic factor
BDNF	Brain derived neurotropic factor
IAPP	Islet amyloid polypeptide
TZD	Thiazolidinediones
PPAR-γ	Peroxisome proliferator-activated receptor gamma
DDP4i	Dipeptidyl peptidase-4 inhibitors
DAT	Dopamine transporter
SGLT2i	Sodium-glucose cotransporter 2 inhibitors
GLP-1 RA	Glucagon-like peptide-1 receptor agonists
